# Two‐Point Change Patterns of LDL‐Cholesterol and hs‐CRP and Risk of Major Adverse Cardiovascular Events in Middle‐Aged and Older Chinese Adults: A Prospective Cohort Study

**DOI:** 10.1002/hsr2.72842

**Published:** 2026-08-06

**Authors:** Jiayin Guo, Bin Zhao, Zongwei Liu, Zhian Liang, Yujun Shen, Xiangchen Dai

**Affiliations:** ^1^ Department of Vascular Surgery Tianjin Medical University General Hospital Tianjin China; ^2^ Department of Pharmacology, Tianjin Key Laboratory of Inflammation Biology Key Laboratory of Immune Microenvironment and Disease (Ministry of Education), School of Basic Medical Sciences, Tianjin Medical University Tianjin China

**Keywords:** aged, cardiovascular diseases, China, cholesterol, cohort studies, C‐reactive protein, LDL, middle aged

## Abstract

**Background and Aims:**

The associations of two‐point change patterns of low‐density lipoprotein cholesterol (LDL‐C) and high‐sensitivity C‐reactive protein (hs‐CRP) with major adverse cardiovascular events (MACE) remain underexplored, particularly in Chinese populations. This study aimed to identify LDL‐C and hs‐CRP change patterns and examine their associations with incident MACE in middle‐aged and older Chinese adults.

**Methods:**

This prospective cohort study included 9597 participants aged 45 years or older and free of cardiovascular disease at baseline from the China Health and Retirement Longitudinal Study. Among them, 6405 participants with biomarker measurements available in both 2011 and 2015 were included in the two‐point change pattern analysis. Group‐based trajectory modeling was used to identify two‐point change pattern groups for LDL‐C and log‐transformed hs‐CRP. Cox proportional hazards models were used to estimate hazard ratios (HRs) and 95% confidence intervals (CIs), with adjustment for potential confounders including lipid‐lowering medication use.

**Results:**

Over a median follow‐up of 9.00 years, 2248 participants (23.4%) developed MACE. Higher baseline hs‐CRP levels were associated with increased MACE risk, with a significant association observed for the fourth quintile versus the first quintile (HR = 1.19, 95% CI: 1.04–1.36). In the two‐point change pattern analysis, the *High baseline, decreasing* log‐hsCRP group showed a lower HR estimate for MACE than the *Low baseline, increasing* group, although this association did not reach conventional statistical significance (HR = 0.82, 95% CI: 0.67–1.01, *p* = 0.06). Neither baseline LDL‐C levels nor LDL‐C two‐point change pattern groups were significantly associated with MACE after full multivariable adjustment. Having one elevated biomarker (LDL‐C Q5 or hs‐CRP Q5) was associated with increased MACE risk (HR = 1.11, 95% CI: 1.02–1.21) compared with having neither elevated.

**Conclusion:**

Baseline hs‐CRP showed a stronger association with MACE than LDL‐C after comprehensive adjustment. A decrease in hs‐CRP from a high baseline level was associated with a lower HR estimate for MACE, although this finding was not statistically significant. These results support the relevance of inflammatory status in cardiovascular risk assessment, while LDL‐C monitoring remains a cornerstone of cardiovascular prevention.

**Abbreviations**
AIC: akaike information criterion
ASCVD: atherosclerotic cardiovascular disease
BIC: Bayesian information criterion
BMI: Body Mass Index
CHARLS: China Health and Retirement Longitudinal Study
CI: confidence interval
GBTM: group‐based trajectory modeling
HDL‐C: high‐density lipoprotein cholesterol
HR: hazard ratio
hs‐CRP: high‐sensitivity C‐reactive protein
IQR: interquartile range
LDL‐C: low‐density lipoprotein cholesterol
Lp(a): lipoprotein(a)
MACE: major adverse cardiovascular events
MICE: multiple imputation by chained equations
Q: quintile
RCS: restricted cubic splines
SD: standard deviation
VLDL‐C: very‐low‐density lipoprotein cholesterol

## 1 Introduction

Cardiovascular diseases represent a leading cause of mortality globally and impose a substantial burden in China, where an increasing incidence is compounded by accelerated population aging and lifestyle transitions, posing significant challenges to the public health system [1, 2]. Low‐density lipoprotein cholesterol (LDL‐C) and chronic low‐grade inflammation, often indicated by high‐sensitivity C‐reactive protein (hs‐CRP), are established key pathophysiological drivers of atherosclerotic cardiovascular disease (ASCVD) [3, 4, 5, 6]. Traditional cardiovascular risk assessment often relies on single‐time measurements of biomarkers such as LDL‐C and hs‐CRP. However, these markers exhibit dynamic changes within individuals over time, and such static “snapshots” may not fully capture the long‐term cardiovascular risk associated with cumulative exposure or evolving patterns [7]. The concept of cumulative exposure to risk factors like LDL‐C has gained prominence, and similarly, cumulative exposure to elevated hs‐CRP has been linked to increased cardiovascular risk [8, 9]. Accordingly, increasing attention has been paid to repeated biomarker measurements and analytical approaches that summarize heterogeneity in biomarker levels and changes over time. One such approach is group‐based trajectory modeling (GBTM), which can be used to identify distinct patterns across repeated observations [10]. However, when only two measurements are available, the resulting groups should be interpreted cautiously, as they primarily reflect baseline level and direction of change between two time points rather than true longitudinal developmental trajectories. Previous studies, predominantly from Western populations, have examined repeated measures or trajectory‐like patterns of LDL‐C or hs‐CRP, with mixed findings [11, 12]. However, there is still a paucity of research from large, representative Chinese cohorts simultaneously examining two‐point change patterns of both LDL‐C and hs‐CRP, their joint biomarker status, and their associations with major adverse cardiovascular events (MACE). This is particularly relevant when considering the widespread use of lipid‐lowering medications, which can significantly modify biomarker levels [13], and complicate the interpretation of biomarker‐MACE associations if not adequately accounted for. Given the potential ethnic and lifestyle differences in cardiovascular risk profiles between Chinese and Western populations [14], and the recognized importance of both inflammatory pathways and lipid metabolism in atherogenesis, a deeper understanding of these relationships within the Chinese context is of significant public health importance. Characterizing two‐point change patterns of LDL‐C and hs‐CRP, together with baseline biomarker levels and joint biomarker elevation, in a large, representative Chinese cohort may improve understanding of their associations with subsequent MACE risk. Importantly, these findings should not be interpreted as diminishing the established importance of LDL‐C monitoring and lipid‐lowering treatment in cardiovascular prevention. Therefore, using prospective data from the China Health and Retirement Longitudinal Study (CHARLS), this study aimed to [1]: identify major GBTM‐derived two‐point change pattern groups of LDL‐C and hs‐CRP among middle‐aged and older Chinese adults based on measurements obtained in 2011 and 2015; and [2] evaluate the associations of these change pattern groups, as well as baseline biomarker levels and joint biomarker elevation, with the risk of incident MACE after adjustment for a comprehensive set of confounders, including lipid‐lowering medication use. We hypothesized that different two‐point change patterns of LDL‐C and hs‐CRP would show differential associations with subsequent MACE risk in this population.

## 2 Methods

### 2.1 Study Design and Data Source

This study employed a prospective cohort design, utilizing data from the CHARLS. CHARLS is an ongoing, nationally representative longitudinal survey that collects comprehensive information on the social, economic, and health status of Chinese adults aged 45 years and older. Baseline interviews for CHARLS commenced in 2011, using a multistage probability sampling method covering 150 county‐level units and 450 village‐level units across China. Participants were followed up approximately every 2 years, with data primarily collected via standardized interviewer‐administered questionnaires. This research used publicly available data from the CHARLS baseline survey (Wave 1) in 2011 through the 2020 follow‐up wave. The Biomedical Ethics Review Committee of Peking University approved the CHARLS protocol (IRB approval number: IRB00001052–11015), and all participants provided written informed consent. Because this study used publicly available, de‐identified CHARLS data, additional ethical approval from our institution was not required.

### 2.2 Study Population

**Figure 1:**
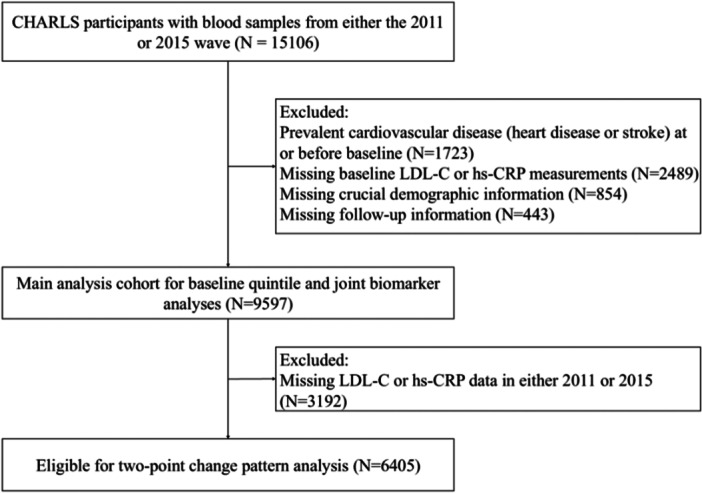
Study flowchart. Flowchart illustrating the participant selection process from the China Health and Retirement Longitudinal Study (CHARLS) for the main analysis cohort and the two‐point change pattern analysis cohort.

The initial target population included 15106 CHARLS participants who underwent blood sample collection in either Wave 1 (2011) or Wave 3 (2015). An individual's baseline for this study was the wave in which they first provided valid blood sample measurements for both LDL‐C and hs‐CRP. Participants were excluded based on the following criteria [1]: self‐reported physician diagnosis of heart disease, encompassing myocardial infarction, coronary heart disease, angina, congestive heart failure, or other heart problems, or stroke at or before their baseline (*N* = 1723) [2]; missing baseline LDL‐C or hs‐CRP measurements (*N* = 2489) [3]; missing crucial demographic information such as age or sex (*N* = 854); or [4] missing follow‐up information, meaning they only had baseline data (*N* = 443). Application of these criteria resulted in a main analysis cohort of 9597 participants, which was used to assess baseline biomarker levels and joint biomarker status in relation to MACE. For the analysis of two‐point change patterns, participants were further required to have valid LDL‐C and hs‐CRP measurements at both Wave 1 (2011) and Wave 3 (2015). This yielded a final two‐point change pattern analysis cohort of 6405 participants after exclusion of 3192 individuals who did not meet these dual‐measurement criteria. Figure 1 details this sample selection process.

### 2.3 Exposure Assessment: LDL‐C and hs‐CRP Levels and Identification of Two‐Point Change Pattern Groups

#### 2.3.1 Baseline Biomarker Levels

Baseline LDL‐C and hs‐CRP levels were determined from blood samples collected at the participant's defined baseline wave. According to CHARLS standard operating procedures, all blood samples for biochemical analysis were collected in the morning after an overnight fast of at least 8 h. Standard enzymatic methods were uniformly used for lipid profile measurements, including LDL‐C. An immunoturbidimetric assay determined hs‐CRP levels. Due to its right‐skewed distribution, hs‐CRP was natural logarithm‐transformed (log(hs‐CRP)) for relevant analyses involving its continuous form and for the analysis of two‐point change pattern groups; a small constant was added to non‐positive hs‐CRP values before transformation. For baseline risk assessment, LDL‐C and hs‐CRP levels were categorized into quintiles (Q1‐Q5).

#### 2.3.2 Identification of Two‐Point Change Pattern Groups

Two‐point change pattern groups of LDL‐C and log‐hsCRP were modeled using measurements from Wave 1 (2011) and Wave 3 (2015). GBTM was performed using the lcmm package (version 2.2.1) in R software (version 4.4.1). Given that only two biomarker measurements were available, these GBTM‐derived groups were interpreted as reflecting baseline level and direction of change between two time points, rather than true longitudinal developmental trajectories. The optimal number of latent classes (ng=2 for both LDL‐C and log‐hsCRP) was determined by systematically exploring models with varying numbers of classes, based on a combination of Bayesian Information Criterion (BIC), Akaike Information Criterion (AIC), model convergence, adequate class size, and the clinical plausibility of the observed two‐point change patterns. Candidate models were evaluated separately for each biomarker by considering alternative class numbers and different placements of covariates in the fixed and mixture components, together with different random‐effects structures. Final model selection was based on a combination of AIC, BIC, convergence, adequate class size, and clinical interpretability. More complex specifications, particularly for log‐hsCRP, showed convergence difficulties, and a simplified final model was therefore retained. The final ng = 2 model for LDL‐C included a linear time effect (wave), adjustments for baseline scaled age and gender in the mixture component (mixture = ~ age_scaled + gender), and a random intercept (random = ~ 1). The final ng = 2 model for log‐hsCRP included a linear time effect, adjustments for baseline scaled age and gender in the fixed effects component, a random intercept (random = ~ 1), and an intercept‐only mixture component (mixture = ~ 1). Because the final selected models differed between biomarkers, the resulting groups should be interpreted as biomarker‐specific GBTM‐derived two‐point change pattern groups rather than directly comparable latent classes. Change pattern group assignment for each participant in the two‐point change pattern cohort was based on their maximum posterior probability of class membership, determined from models fitted to each of the five imputed datasets, with a majority vote rule applied. The identified groups were descriptively labeled as *Low baseline, increasing* and *High baseline, decreasing* based on their observed patterns.

### 2.4 Outcome Assessment

The primary outcome was incident MACE, defined as the first occurrence of self‐reported, physician‐diagnosed heart disease (myocardial infarction, coronary heart disease, angina, congestive heart failure, or other heart problems) or stroke after the baseline wave. Event occurrence and timing were ascertained from follow‐up questionnaires administered approximately every 2 years until 2020. Follow‐up time was calculated in years from each participant's baseline survey year to the year of their first MACE event, or to their last valid follow‐up survey year if no MACE occurred. Because outcome ascertainment relied on self‐report of physician diagnosis, outcome misclassification could not be fully excluded.

### 2.5 Covariate Assessment

Comprehensive information on potential confounding factors was collected at baseline. The following covariates were included in multivariable‐adjusted models: age (years), gender, education level, residence, marital status, current smoking status, current drinking status, regular physical activity, body mass index (BMI, kg/m^2^), hypertension, diabetes, self‐rated health, retirement status, household consumption per capita (Yuan/year), household income total (Yuan/year), and use of lipid‐lowering medication. Original CHARLS coding was used where feasible, and invalid responses were treated as missing before imputation.

### 2.6 Handling of Missing Data

Missing data for baseline covariates (detailed proportions in Supplementary Table S1) were addressed using multiple imputation by chained equations (MICE). This was implemented with the IterativeImputer function (employing Bayesian ridge regression) from the scikit‐learn package (version 1.6.1) in Python (version 3.13.0). The imputation model included all covariates, baseline LDL‐C and hs‐CRP levels, MACE status, and follow‐up time. Five complete imputed datasets (*m* = 5) were generated. BMI values outside the range of 10–70 kg/m^2^ were set to missing before imputation.

### 2.7 Statistical Analysis

Baseline characteristics were described using means ± standard deviation (SD) or medians (interquartile range, IQR) for continuous variables, and frequencies (percentages) for categorical variables. Differences between MACE and no MACE groups were assessed using *t*‐tests or Mann‐Whitney U tests for continuous variables, and chi‐square tests for categorical variables. Multivariable‐adjusted Cox proportional hazards models estimated hazard ratios (HRs) and 95% confidence intervals (CIs) for the association of baseline biomarker quintiles (Q1 as reference) and two‐point change pattern groups (Group 1: *Low baseline, increasing* as reference) with incident MACE. All primary Cox models were adjusted for the full prespecified covariate set described above. The proportional hazards assumption was evaluated using Schoenfeld residuals. Restricted cubic splines (RCS) with four knots (at the 5%, 35%, 65%, and 95%) were used to model dose‐response relationships for continuous baseline LDL‐C and baseline log(hs‐CRP) with MACE risk, providing *p* values for overall association and non‐linearity. These RCS analyses were based on baseline biomarker levels only and were not intended to assess longitudinal or time‐varying non‐linearity. Predefined exploratory subgroup analyses were conducted for the associations of baseline hs‐CRP (Q5 vs. Q1) and log‐hsCRP two‐point change pattern groups with MACE, stratified by age, gender, BMI, diabetes, hypertension, residence, smoking status, education, marital status, drinking status, physical activity, self‐rated health, retirement status, and lipid‐lowering medication use. Cox models within subgroups were adjusted for a reduced set of confounders including age, gender, BMI, hypertension, diabetes, lipid‐lowering medication, smoking status, and residence, excluding the stratification variable itself. *p* values for interaction were obtained by including a product term between the main exposure and the subgroup variable in a Cox model fitted to the entire cohort, adjusted for the reduced confounder set. The “Overall” estimates in forest plots for subgroup analyses were derived from models adjusted for the full set of confounders applied to the entire respective analysis cohort. All results from analyses on the five imputed datasets were pooled using Rubin's rules. Analyses were performed using Python (version 3.13.0) with the pandas, numpy, scikit‐learn (version 1.6.1), and lifelines (version 0.30.0) packages, and R (version 4.4.1) with the lcmm (version 2.2.1) and tidyverse packages. All statistical tests were two‐sided, with a two‐sided significance threshold of *p* < 0.05.

## 3 Results

### 3.1 Baseline Characteristics of the Study Population

The study flowchart (Figure 1) illustrates the participant selection process. The main analysis cohort included 9597 participants from the CHARLS who were free of cardiovascular disease at baseline. Among these, 6405 individuals with complete biomarker measurements at both Wave 1 (2011) and Wave 3 (2015) formed the two‐point change pattern analysis cohort. The median follow‐up duration for the main analysis cohort was 9.00 years (IQR, 7.00–9.00). During this period, 2248 (23.4%) participants developed incident MACE.

Table 1 presents the baseline characteristics of the main analysis cohort, stratified by MACE status. Participants who developed MACE were, on average, older (mean 59.94 vs. 57.93 years, *p* < 0.001) and had a higher mean BMI (24.08 vs. 23.20 kg/m^2^, *p*< 0.001). Baseline LDL‐C levels (mean 118.55 vs. 114.79 mg/dL, *p* = 0.001) and hs‐CRP levels (median 1.12 vs. 0.94 mg/L, *p* < 0.001) were also higher in the MACE group. The median follow‐up time was shorter for participants who developed MACE (7.00 vs. 9.00 years, *p* < 0.001). Significant differences (*p* < 0.05) between groups were also observed for gender, education level, marital status, self‐rated health, retirement status, and the use of lipid‐lowering medication (Table 1).

**Table 1:** Baseline characteristics of the study population (*N* = 9597).

Characteristic	Overall	No MACE	MACE	*p* value
Total sample size, *N*	9597	7349	2248	
Age, years	58.40 ± 9.65	57.93 ± 9.79	59.94 ± 9.02	*p* < 0.001
BMI, kg/m^2^	23.40 ± 3.78	23.20 ± 3.70	24.08 ± 3.98	*p* < 0.001
LDL‐C, mg/dL	115.67 ± 34.59	114.79 ± 34.31	118.55 ± 35.37	*p* < 0.001
hs‐CRP, mg/L	0.98 (0.53‐2.08)	0.94 (0.52‐1.99)	1.12 (0.59‐2.30)	*p* < 0.001
Household consumption per capita, yuan	4537.88 (2657.83‐8009.79)	4537.88 (2638.89‐8049.24)	4535.98 (2743.06‐7925.51)	*p* = 0.88
Total household income, yuan	10416.67 (2234.85‐30321.97)	10700.76 (2272.73‐31136.36)	9571.97 (2045.45‐27978.22)	*p* = 0.05
Follow‐up time, years	9.00 (7.00‐9.00)	9.00 (9.00‐9.00)	7.00 (4.00‐9.00)	*p* < 0.001
Gender				*p* < 0.001
Female	5085 (53.0%)	3796 (51.7%)	1289 (57.3%)	
Male	4512 (47.0%)	3553 (48.3%)	959 (42.7%)	
Residence				*p* = 0.56
Rural	3352 (34.9%)	2579 (35.1%)	773 (34.4%)	
Urban	6245 (65.1%)	4770 (64.9%)	1475 (65.6%)	
Education level				*p* = 0.009
Primary school	4796 (50.0%)	3624 (49.3%)	1172 (52.1%)	
Junior high school	1975 (20.6%)	1508 (20.5%)	467 (20.8%)	
Senior high school	1838 (19.2%)	1461 (19.9%)	377 (16.8%)	
College and above	988 (10.3%)	756 (10.3%)	232 (10.3%)	
Marital status				*p* = 0.04
Married/Cohabiting	7987 (83.3%)	6139 (83.6%)	1848 (82.3%)	
Separated	517 (5.4%)	412 (5.6%)	105 (4.7%)	
Divorced	0 (0.0%)	0 (0.0%)	0 (0.0%)	
Widowed	46 (0.5%)	33 (0.4%)	13 (0.6%)	
Never married	58 (0.6%)	42 (0.6%)	16 (0.7%)	
Domestic partnership	0 (0.0%)	0 (0.0%)	0 (0.0%)	
Other	902 (9.4%)	656 (8.9%)	246 (11.0%)	
Unknown	80 (0.8%)	62 (0.8%)	18 (0.8%)	
Self‐rated health				*p* < 0.001
Excellent	346 (3.6%)	230 (3.1%)	116 (5.2%)	
Very good	1991 (20.8%)	1347 (18.4%)	644 (28.8%)	
Good	4904 (51.3%)	3796 (51.8%)	1108 (49.6%)	
Fair	1722 (18.0%)	1445 (19.7%)	277 (12.4%)	
Poor	593 (6.2%)	504 (6.9%)	89 (4.0%)	
Retirement status				*p* = 0.04
No	8478 (90.7%)	6529 (91.0%)	1949 (89.5%)	
Yes	874 (9.3%)	645 (9.0%)	229 (10.5%)	
Lipid‐lowering medication				*p* < 0.001
No	9019 (96.4%)	6972 (97.3%)	2047 (93.5%)	
Yes	336 (3.6%)	193 (2.7%)	143 (6.5%)	

*Note:* Data are presented as mean ± standard deviation (SD) for approximately normally distributed continuous variables, median (interquartile range, [IQR]) for skewed continuous variables, or *n* (%) for categorical variables. *p* values were derived from Student's t‐tests or Mann‐Whitney U tests for continuous variables and chi‐square tests for categorical variables, comparing participants who developed MACE versus those who did not. Some categorical variables are presented here in their original descriptive categories but were collapsed into broader categories for multivariable analyses, as described in the Methods. Regular physical activity: “Yes” indicates engaging in regular physical activity as defined in the CHARLS survey. Lipid‐lowering medication was based on self‐report of current use at baseline.

Abbreviations: BMI, Body Mass Index; hs‐CRP, high‐sensitivity C‐reactive protein; LDL‐C, low‐density lipoprotein cholesterol; MACE, Major Adverse Cardiovascular Events.

### 3.2 Identification and Characteristics of LDL‐C and Log‐hsCRP Two‐Point Change Pattern Groups

**Figure 2:**
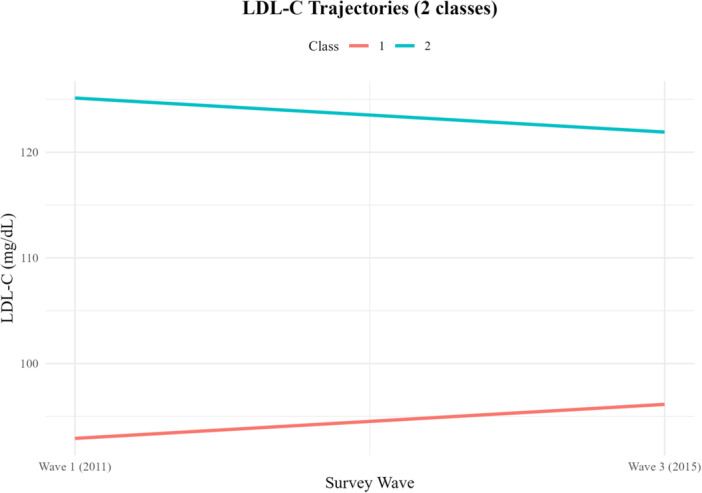
GBTM‐derived two‐point change pattern groups of LDL‐C. Two GBTM‐derived two‐point change pattern groups of LDL‐C (mg/dL) were identified among 6405 participants with measurements at Wave 1 (2011) and Wave 3 (2015). Group 1: *Low baseline, increasing* (*N* = 174, 2.7%); Group 2: *High baseline, decreasing* (*N* = 6227, 97.3%). Lines represent mean predicted LDL‐C levels for each group based on the two available measurements. With only two time points, these groups reflect baseline level and direction of change rather than true longitudinal developmental trajectories.

**Figure 3:**
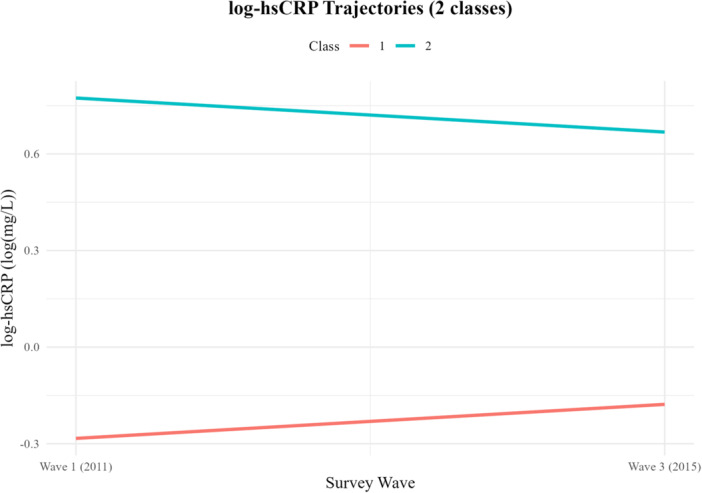
GBTM‐derived two‐point change pattern groups of log(hs‐CRP). Two GBTM‐derived two‐point change pattern groups of log‐transformed hs‐CRP were identified among 6405 participants with measurements at Wave 1 (2011) and Wave 3 (2015). Group 1: *Low baseline, increasing* (*N* = 323, 5.0%); Group 2: *High baseline, decreasing* (*N* = 6078, 95.0%). Lines represent mean predicted log(hs‐CRP) levels for each group based on the two available measurements. With only two time points, these groups reflect baseline level and direction of change rather than true longitudinal developmental trajectories.

Among the 6405 participants included in the two‐point change pattern analysis, GBTM identified two distinct two‐point change pattern groups for LDL‐C and two for log‐hsCRP based on measurements from 2011 to 2015. For LDL‐C (Figure 2), Group 1, labeled *Low baseline, increasing*, comprised 174 participants (2.7%) and was characterized by lower baseline LDL‐C levels with a slight increase between the two measurements. The majority of participants were classified into Group 2, labeled *High baseline, decreasing* (*n* = 6227, 97.3%), which showed higher baseline LDL‐C levels with a slight decrease between 2011 and 2015. For log‐hsCRP (Figure 3), Group 1, labeled *Low baseline, increasing* (*n* = 323, 5.0%), was characterized by lower baseline log‐hsCRP levels followed by an increase between the two measurements. Group 2, labeled *High baseline, decreasing* (*n* = 6078, 95.0%), showed higher baseline log‐hsCRP levels followed by a decrease between 2011 and 2015. Given that only two biomarker measurements were available and that the identified groups were highly unbalanced in size, these groups are presented as GBTM‐derived two‐point change pattern groups rather than true longitudinal developmental trajectories. Baseline characteristics were not formally compared between groups.

### 3.3 Associations of Baseline Biomarker Levels With MACE Risk

**Figure 4:**
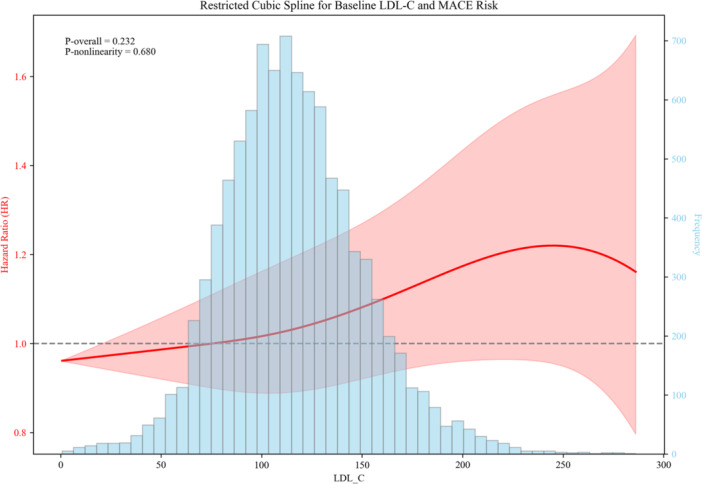
Association between baseline LDL‐C and risk of MACE using restricted cubic splines. Multivariable‐adjusted hazard ratios (HRs) and 95% confidence intervals (CIs) for major adverse cardiovascular events (MACE) according to continuous baseline LDL‐C levels. The solid line represents the estimated HR, and the shaded area represents the 95% CI. The dashed horizontal line indicates the reference value (HR = 1.0). Knots were placed at the 5th, 35th, 65th, and 95th percentiles of the LDL‐C distribution. These restricted cubic spline analyses were based on baseline LDL‐C levels only. *p* value for overall association = 0.23; *p* value for non‐linearity = 0.68.

After multivariable adjustment, including for lipid‐lowering medication, baseline LDL‐C levels categorized by quintiles were not significantly associated with MACE risk. Compared to the lowest quintile (Q1), the hazard ratios (HRs) for MACE for quintiles Q2 through Q5 were all non‐significant (all *p* > 0.05; Table 2). Restricted cubic spline analysis of continuous baseline LDL‐C levels further showed no significant overall association with MACE risk (P for overall association = 0.23) and no evidence of a non‐linear relationship (P for non‐linearity = 0.68, Figure 4).

**Table 2:** Associations of baseline LDL‐C with risk of incident MACE (*N* = 9597).

Quintile	N	Events	HR (95% CI)	*p* value
Q1	1923	394	1.00 (Reference)	Ref.
Q2	1954	428	1.00 (0.87–1.15)	*p* > 0.99
Q3	1885	458	1.10 (0.96–1.26)	*p* = 0.17
Q4	1916	463	1.06 (0.93–1.22)	*p* = 0.36
Q5	1919	505	1.09 (0.96–1.25)	*p* = 0.19

*Note:* Models were adjusted for the full prespecified covariate set, including age, gender, education level, residence, marital status, current smoking status, current drinking status, regular physical activity, Body Mass Index, hypertension, diabetes, self‐rated health, retirement status, household consumption per capita, household income total, and use of lipid‐lowering medication. LDL‐C quintiles (Q1–Q5) for LDL‐C were defined according to the baseline distribution in the study population.

Abbreviations: CI, confidence interval; HR, hazard ratio; LDL‐C, low‐density lipoprotein cholesterol; MACE, Major Adverse Cardiovascular Events; Q, Quintile.

**Figure 5:**
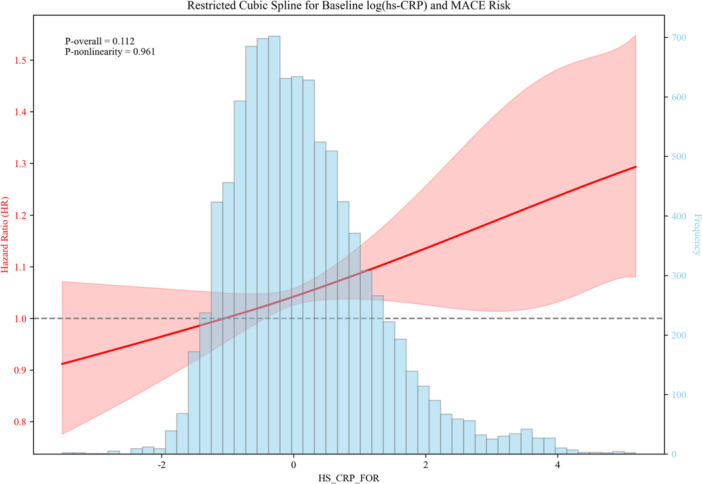
Association between baseline log(hs‐CRP) and risk of MACE using restricted cubic splines. Multivariable‐adjusted hazard ratios (HRs) and 95% confidence intervals (CIs) for major adverse cardiovascular events (MACE) according to continuous baseline log‐transformed hs‐CRP levels. The solid line represents the estimated HR, and the shaded area represents the 95% CI. The dashed horizontal line indicates the reference value (HR = 1.0). Knots were placed at the 5%, 35%, 65%, and 95% of the log(hs‐CRP) distribution. These restricted cubic spline analyses were based on baseline log(hs‐CRP) levels only. *p* value for overall association = 0.11; *p* value for non‐linearity = 0.96.

In contrast, higher baseline hs‐CRP levels were associated with an increased MACE risk. Participants in the fourth quintile (Q4) of hs‐CRP had a significantly higher risk of MACE compared to those in the lowest quintile (Q1) (HR = 1.19, 95% CI: 1.04–1.36, *p* = 0.01). For the highest quintile (Q5), the association did not reach statistical significance (HR = 1.11, 95% CI: 0.97–1.28, *p*= 0.13) (Table 3). In the restricted cubic spline analysis of baseline log‐transformed hs‐CRP, the overall association with MACE risk did not reach statistical significance (P for overall association = 0.11), and there was no evidence of non‐linearity (P for non‐linearity = 0.96, Figure 5). Visually, the spline suggested a generally increasing pattern across higher baseline log(hs‐CRP) values, but this finding should be interpreted cautiously given the non‐significant overall test.

**Table 3:** Associations of baseline hs‐CRP Quintiles with Risk of Incident MACE (*N* = 9597).

Quintile	*N*	Events	HR (95% CI)	*p* value
Q1	1966	381	1.00 (Reference)	Ref.
Q2	1916	404	1.02 (0.88–1.17)	*p* = 0.82
Q3	1880	459	1.12 (0.98–1.28)	*p* = 0.11
Q4	1915	509	1.19 (1.04–1.36)	*p* = 0.01
Q5	1920	495	1.11 (0.97–1.28)	*p* = 0.13

*Note:* Models were adjusted for the full prespecified covariate set, including age, gender, education level, residence, marital status, current smoking status, current drinking status, regular physical activity, Body Mass Index, hypertension, diabetes, self‐rated health, retirement status, household consumption per capita, household income total, and use of lipid‐lowering medication. hs‐CRP quintiles (Q1–Q5) for hs‐CRP were defined according to the baseline distribution in the study population.

Abbreviations: CI, confidence interval; HR, hazard ratio; LDL‐C, low‐density lipoprotein cholesterol; MACE, Major Adverse Cardiovascular Events; Q, Quintile.

### 3.4 Joint Association of High LDL‐C and High hs‐CRP Levels With Mace Risk

The combined effect of high baseline LDL‐C, defined as quintile 5, and high hs‐CRP, also quintile 5, on MACE risk is shown in Table 4. Compared to participants with neither biomarker at a high level, those with one high biomarker, either high LDL‐C or high hs‐CRP, had a significantly increased risk of MACE; the hazard ratio was 1.11 (95% CI: 1.02–1.21, *p* = 0.02). However, participants with both biomarkers at high levels did not show a further statistically significant increase in MACE risk when compared to the group with no high biomarkers; their hazard ratio was 0.90 (95% CI: 0.73–1.10, *p* = 0.31). Thus, having one elevated biomarker was associated with higher MACE risk, whereas the group with concurrent elevation of both biomarkers did not show a statistically significant association in this analysis.

**Table 4:** Association of number of elevated biomarkers (LDL‐C Q5 or hs‐CRP Q5) with risk of incident MACE (*N* = 9597).

Number of elevated biomarkers	*N*	Events	HR (95% CI)	*p* value
0 (Reference)	6178	1349	1.00 (Reference)	Ref.
1	2999	798	1.11 (1.02–1.21)	*p* = 0.02
2	420	101	0.90 (0.73–1.10)	*p* = 0.31

*Note:* High LDL‐C was defined as in Q5 of the baseline LDL‐C distribution. High hs‐CRP was defined as hs‐CRP in Q5 of the baseline hs‐CRP distribution. Number of High‐Level Biomarkers = 0 indicates LDL‐C not in Q5 AND hs‐CRP not in Q5. Number of High‐Level Biomarkers = 1 indicates either LDL‐C in Q5 OR hs‐CRP in Q5, but not both. Number of High‐Level Biomarkers = 2 indicates LDL‐C in Q5 AND hs‐CRP in Q5. Models were adjusted for the full prespecified covariate set, including age, gender, education level, residence, marital status, current smoking status, current drinking status, regular physical activity, Body Mass Index, hypertension, diabetes, self‐rated health, retirement status, household consumption per capita, household income total, and use of lipid‐lowering medication.

Abbreviations: CI, confidence interval; HR, hazard ratio; LDL‐C, low‐density lipoprotein cholesterol; MACE, Major Adverse Cardiovascular Events; Q, Quintile.

### 3.5 Associations of Two‐Point Change Pattern Groups With MACE Risk

After multivariable adjustment, the LDL‐C two‐point change pattern groups were not significantly associated with MACE risk. Compared to Group 1 (*Low baseline, increasing*), the HR for Group 2 (*High baseline, decreasing*) was 0.80 (95% CI: 0.61–1.05, *p* = 0.11). The small sample size of Group 1 in the LDL‐C two‐point change pattern analysis (N = 174) should be considered when interpreting this result.

For log‐hsCRP two‐point change pattern groups, Group 2 (*High baseline, decreasing*) showed a lower HR estimate for MACE compared to Group 1 (*Low baseline, increasing*), which served as the reference group (HR = 0.82, 95% CI: 0.67–1.01, *p* = 0.06) (Table 5). However, this association did not reach conventional statistical significance and should be interpreted cautiously.

**Table 5:** Associations of log‐hsCRP two‐point change pattern groupswith risk of incident MACE (*N* = 6405).

log‐hsCRP two‐point change pattern group	*N*	Events	HR (95% CI)	*p* value
Low baseline, increasing (Reference)	323	50	1.00 (Reference)	Ref.
High baseline, decreasing	6078	500	0.82 (0.67‐1.01)	*p*= 0.06

*Note:* Group 1: Low baseline, increasing log‐hsCRP two‐point change pattern group. Group 2: High baseline, decreasing two‐point change pattern group. Models were adjusted for the full prespecified covariate set, including age, gender, education level, residence, marital status, current smoking status, current drinking status, regular physical activity, Body Mass Index, hypertension, diabetes, self‐rated health, retirement status, household consumption per capita, household income total, and use of lipid‐lowering medication. Because the LDL‐C two‐point change pattern groups were highly imbalanced class in size, for LDL‐C two‐point change pattern groups, the corresponding detailed results are described in the main text rather than presented as a separate table.

Abbreviations: CI, confidence interval; HR, hazard ratio; hs‐CRP, high‐sensitivity C‐reactive protein; LDL‐C, low‐density lipoprotein cholesterol; MACE, Major Adverse Cardiovascular Events.

### 3.6 Exploratory Subgroup Analyses

#### 3.6.1 Baseline hs‐CRP (Q5 vs. Q1)

**Figure 6:**
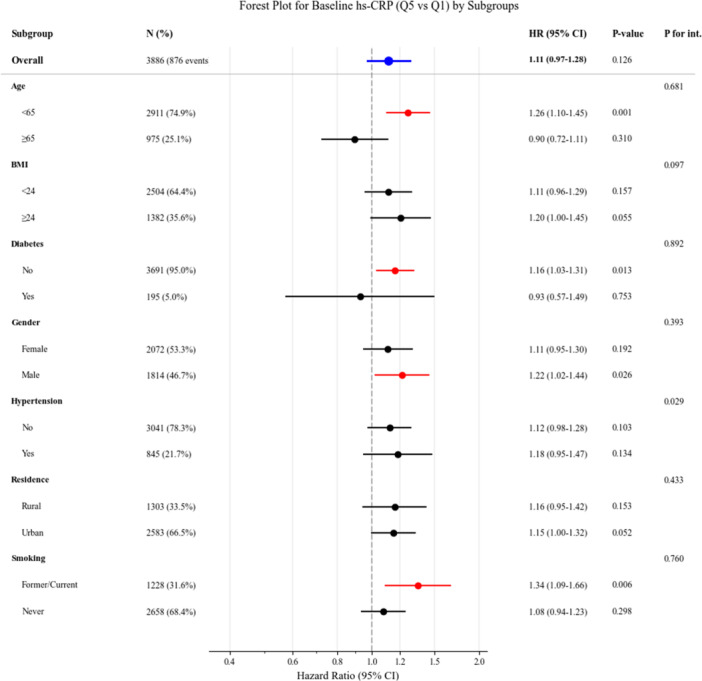
Exploratory subgroup analyses for the association between baseline hs‐CRP (Q5 vs. Q1) and incident MACE. Forest plot showing exploratory subgroup analyses of the association between baseline hs‐CRP (Q5 vs. Q1) and incident major adverse cardiovascular events (MACE). The overall estimate was adjusted for the full prespecified covariate set. Subgroup analyses were adjusted for age, gender, BMI, hypertension, diabetes, lipid‐lowering medication use, smoking status, and residence, excluding the stratification variable itself. *p* values for interaction are shown. Because the overall association did not reach conventional statistical significance, these subgroup findings should be interpreted cautiously.

The association between high baseline hs‐CRP (Q5 vs. Q1) and MACE risk was examined across predefined subgroups **(**Figure 6 **)**. The overall adjusted HR was 1.11 (95% CI: 0.97‐1.28, *p* = 0.13). A statistically significant interaction was observed with hypertension status (P for interaction = 0.03). Specifically, the HR for MACE among participants with hypertension was 1.18 (95% CI: 0.95‐1.47, *p* = 0.13), while in those without hypertension, the HR was 1.12 (95% CI: 0.98‐1.28, *p* = 0.10). Potential interactions were also suggested for age (< 65 vs. ≥ 65 years, P for interaction = 0.08), BMI (< 24 vs. ≥ 24 kg/m^2^, P for interaction = 0.10), and diabetes status (P for interaction = 0.09). No significant interactions were found for gender, residence, or smoking status. Because the overall association between baseline hs‐CRP (Q5 vs. Q1) and MACE did not reach statistical significance, these subgroup findings should be interpreted as exploratory.

#### 3.6.2 log‐hsCRP Two‐Point Change Pattern Groups (High Baseline, Decreasing vs. Low Baseline, Increasing)

**Figure 7:**
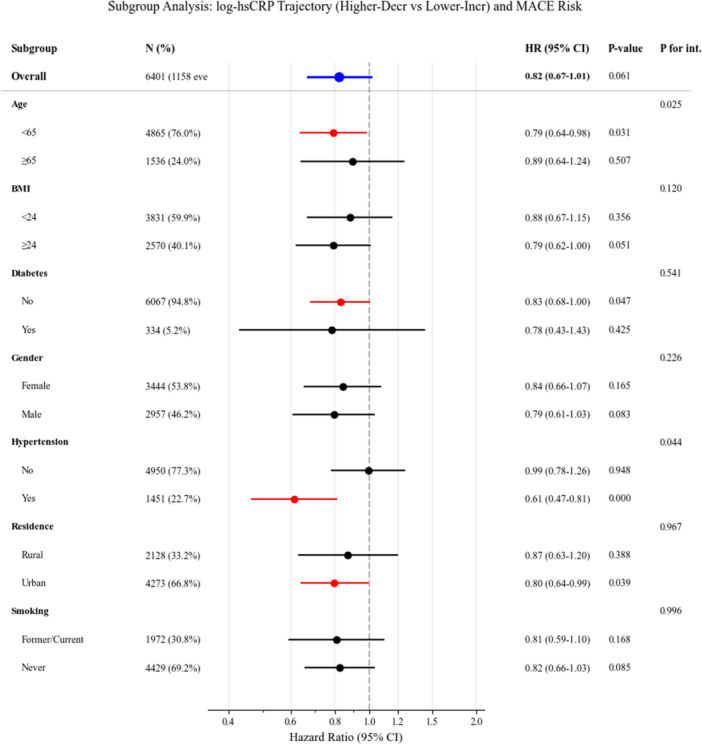
Exploratory subgroup analyses for the association between log‐hsCRP two‐point change pattern groups and incident MACE. Forest plot showing exploratory subgroup analyses of the association between the *High baseline, decreasing* and *Low baseline, increasing* log‐hsCRP two‐point change pattern groups and incident major adverse cardiovascular events (MACE). The overall estimate was adjusted for the full prespecified covariate set. Subgroup analyses were adjusted for age, gender, BMI, hypertension, diabetes, lipid‐lowering medication use, smoking status, and residence, excluding the stratification variable itself. *p* values for interaction are shown. Because the overall association did not reach conventional statistical significance, these subgroup findings should be interpreted cautiously.

The association of the *High baseline, decreasing* log‐hsCRP two‐point change pattern group with MACE risk was further explored in subgroups (Figure 7). The overall adjusted HR was 0.82 (95% CI: 0.67–1.01, *p* = 0.06). This association appeared to differ by age (P for interaction = 0.03) and hypertension status (P for interaction = 0.04). Specifically, the inverse association was more evident in participants younger than 65 years (HR = 0.79, 95% CI: 0.64–0.98) and in those with hypertension (HR = 0.61, 95% CI: 0.47–0.81). No statistically significant interactions were detected for BMI, diabetes, gender, residence, or smoking status. Given that the overall association did not reach conventional statistical significance, these subgroup results should also be interpreted cautiously and as exploratory rather than confirmatory.

## 4 Discussion

### 4.1 Summary of Main Findings

In this large prospective cohort study of 9597 middle‐aged and older Chinese adults free of cardiovascular disease at baseline and followed for a median of 9.00 years, we examined the associations of baseline levels and GBTM‐derived two‐point change pattern groups of LDL‐C and hs‐CRP with incident MACE. Higher baseline hs‐CRP levels, particularly the fourth quintile, were significantly associated with an increased MACE risk. In the log‐hsCRP two‐point change pattern analysis, the group with a high baseline level followed by a decrease had a lower HR estimate for MACE than the group with a low baseline level followed by an increase, although this association did not reach conventional statistical significance (HR = 0.82, *p*= 0.06). In exploratory subgroup analyses, this inverse association appeared more evident among participants younger than 65 years and those with hypertension, but these findings should be interpreted with caution. By contrast, after comprehensive multivariable adjustment, including lipid‐lowering medication use, neither baseline LDL‐C levels nor LDL‐C two‐point change pattern groups were significantly associated with MACE. We also found that having one elevated biomarker, either LDL‐C Q5 or hs‐CRP Q5, was associated with increased MACE risk compared with having neither elevated, whereas concurrent elevation of both biomarkers was not associated with a statistically significant increase in risk.

### 4.2 Interpretation of Findings in Context of Existing Literature

The main finding of this study is that baseline hs‐CRP, particularly at moderately to highly elevated levels, showed a stronger association with subsequent MACE risk than baseline LDL‐C after multivariable adjustment. The log‐hsCRP two‐point change pattern analysis suggested that a decrease from a high baseline level may be associated with lower subsequent risk than an increase from a low baseline level, although this finding did not reach conventional statistical significance. In contrast, neither baseline LDL‐C quintiles nor LDL‐C two‐point change pattern groups showed significant associations with MACE after adjustment, including adjustment for lipid‐lowering medication use. These findings should not be interpreted as diminishing the established importance of LDL‐C monitoring and lipid‐lowering treatment in cardiovascular prevention. The association between higher baseline hs‐CRP levels, particularly Q4, and increased MACE risk is consistent with a substantial body of evidence supporting inflammation as a key pathological process in the development and progression of atherosclerosis and cardiovascular events [6, 15]. For example, Lee et al. reported in a Korean cohort that early elevation of hs‐CRP was associated with cardiovascular disease incidence and all‐cause mortality [16]. Similarly, Kraaijenhof et al. in the EPIC‐Norfolk study showed that increasing quintiles of baseline hs‐CRP were associated with higher 20‐year MACE risk [5]. In our restricted cubic spline analysis, the overall association between baseline log(hs‐CRP) and MACE did not reach statistical significance, and no evidence of non‐linearity was observed. Still, the visual pattern was broadly consistent with higher risk at higher baseline hs‐CRP levels, in line with previous literature supporting hs‐CRP as a marker of residual inflammatory risk [15]. Although Q4 was significantly associated with MACE, Q5 was not. The two quintiles were similar in size (Q4: *n* = 1915; Q5: *n* = 1920) and had comparable numbers of events (Q4: 509; Q5: 495). In addition, the confidence intervals overlapped substantially. These features suggest that the isolated statistical significance in Q4 should be interpreted cautiously and may reflect modest estimate instability around category cut‐points rather than a robust threshold effect. Consistent with this interpretation, the spline analysis did not support clear non‐linearity across baseline log(hs‐CRP) levels.

A more tentative finding concerned the log‐hsCRP two‐point change pattern groups. Compared with the *Low baseline, increasing* group, the *High baseline, decreasing* group had a lower HR estimate for MACE. However, the confidence interval crossed 1, and the overall association did not reach conventional statistical significance. This result should therefore be interpreted as suggestive rather than confirmatory. One possible explanation is that improvement in inflammatory status between the two measurement occasions may be linked to lower subsequent cardiovascular risk, even among individuals starting from a higher inflammatory state. This interpretation is directionally consistent with prior studies showing that cumulative exposure to elevated hs‐CRP is associated with adverse cardiovascular outcomes [8] and that follow‐up hs‐CRP measurements may provide clinically relevant information beyond a single baseline value [17]. At the same time, because our analysis relied on only two time points and the identified groups were highly unbalanced in size, these groups mainly reflect baseline level and direction of change rather than true longitudinal developmental patterns. Therefore, the corresponding findings should be regarded as exploratory and hypothesis‐generating rather than definitive evidence of distinct biological trajectories.

The subgroup findings for age and hypertension status also require caution. Although the inverse association for the *High baseline, decreasing* log‐hsCRP group appeared more evident among participants younger than 65 years and those with hypertension, the overall association for the main comparison did not reach conventional statistical significance, and the subgroup analyses were exploratory. These patterns may reflect differences in baseline cardiovascular burden, treatment intensity, residual inflammatory risk, or simple chance, and they require confirmation in future studies with denser repeated biomarker measurements.

In contrast to hs‐CRP, we did not observe a significant independent association between baseline LDL‐C quintiles and MACE after comprehensive multivariable adjustment, including lipid‐lowering medication use. Likewise, the two identified LDL‐C two‐point change pattern groups, *Low baseline, increasing* and *High baseline, decreasing*, were not significantly associated with MACE in this cohort. This finding appears to differ from a substantial body of evidence, much of it from Western populations, showing that LDL‐C is a major treatment target and that cumulative LDL‐C exposure is strongly linked to ASCVD risk, often summarized as “lower for longer is better” [3, 7, 9]. Several factors may help explain this discrepancy in our Chinese cohort. First, adjustment for lipid‐lowering medication use may have attenuated the observable association between LDL‐C and MACE. Although only 3.6% of participants reported lipid‐lowering medication use at baseline, use may have increased during follow‐up or been concentrated among participants with high LDL‐C. Individuals with elevated LDL‐C are more likely to receive statins, which reduce both LDL‐C and cardiovascular risk and may also affect hs‐CRP levels [13]. In an observational setting, this can weaken the apparent association between LDL‐C and outcomes. Second, the association of LDL‐C with cardiovascular risk may vary across ethnicities and populations. Naito et al. described racial differences in the cholesterol‐lowering effect of statins between Asian and Western patients, and a meta‐analysis by Li et al. suggested that Asians may respond to lower statin doses than Westerners in terms of coronary plaque regression [14, 18]. In addition, some studies in Asian populations have suggested that other lipid‐related factors, such as LDL particle size or oxidation status, may contribute importantly to risk. For example, Ikezaki et al. reported that small dense LDL‐C may be a more atherogenic lipoprotein parameter [19]. The 2023 Chinese guideline for lipid management still recommends LDL‐C as the primary target for lipid control [4], making our findings on its limited independent association in this analysis after medication adjustment particularly relevant for discussion within the Chinese context, especially given reports like Zhang et al. [20] on the prevalence of dyslipidemia and challenges in achieving LDL‐C targets in Chinese adults. Third, using only two time points for change pattern analysis may not have captured the full complexity of long‐term LDL‐C variability and its association with MACE, especially because 97.3% of participants fell into one LDL‐C group [10]. The small size of the *Low baseline, increasing* LDL‐C group (*N* = 174) also limits the statistical power.

Our joint analysis showed that having one high biomarker, either LDL‐C Q5 or hs‐CRP Q5, was associated with an approximately 11% higher risk of MACE compared with having neither elevated. By contrast, having both biomarkers elevated was not associated with a further statistically significant increase in risk. This somewhat counterintuitive finding should be interpreted cautiously. The two‐high subgroup was relatively small (*N* = 420, with 101 events), which may have reduced precision after subgrouping. In addition, the effect of high LDL‐C may have been attenuated after full adjustment, particularly in the context of treatment‐related factors, and residual confounding or estimate instability cannot be excluded. Therefore, the discrepancy between the one‐high and two‐high groups is more likely to reflect limited precision, treatment‐related influences, or chance than to suggest that concurrent elevation of both biomarkers is clinically unimportant. Taken together, the baseline quintile analysis, spline analysis, joint biomarker analysis, and two‐point change pattern analysis address related but not identical questions. The overall pattern of results suggests that baseline hs‐CRP showed a more consistent association signal with MACE than LDL‐C in this cohort, whereas the findings from the joint biomarker and two‐point change pattern analyses are more exploratory and should be interpreted in light of limited precision, subgroup imbalance, and the constraints of having only two repeated measurements.

### 4.3 Strengths of the Study

This study has several strengths. It was based on the CHARLS, a large, nationally representative prospective cohort of middle‐aged and older Chinese adults, which enhances the relevance of our findings to this population. We evaluated both baseline levels and GBTM‐derived two‐point change pattern groups of LDL‐C and hs‐CRP in the same study. Another strength is the comprehensive adjustment for a wide range of potential confounders, including lipid‐lowering medication use, which is important when interpreting biomarker associations in observational research. The use of multiple imputation also improved the robustness of the analyses. In addition, subgroup analyses and restricted cubic splines provide complementary information on potential effect modification and dose‐response patterns, although the subgroup findings should be regarded as exploratory.

### 4.4 Limitations of the Study

Several limitations should be acknowledged. First, the two‐point change pattern analysis was based on only two time points, 2011 and 2015. As discussed by Nagin and Odgers, this limits the ability to capture more complex and potentially non‐linear patterns of biomarker change over time; three or more repeated measurements would be preferable for more robust group identification and a fuller understanding of longitudinal dynamics [10]. Second, for LDL‐C, one group (*Low baseline, increasing*) was much smaller than the other, which limited statistical power and reduced the stability of the association estimates. Third, MACE outcomes were based on self‐report, albeit prompted by physician diagnosis, and may therefore be subject to recall or misclassification bias compared with adjudicated event data. Fourth, despite adjusting for many covariates, residual confounding from unmeasured or imprecisely measured factors, including medication types, dosages, duration, and adherence, cannot be excluded. Changes in lipid‐lowering or other cardiometabolic treatments between 2011 and 2015 may also have influenced both biomarker levels and subsequent cardiovascular risk. Fifth, hs‐CRP may be influenced by acute intercurrent illnesses, and two measurements may still not adequately capture an individual's long‐term inflammatory state. Single biomarker assessments at each wave may also have been influenced by short‐term biological or measurement variability. Finally, our findings are specific to middle‐aged and older Chinese adults in CHARLS and may not be directly generalizable to other populations. Because this was an observational study, reverse causality cannot be fully excluded, particularly for hs‐CRP, which may partly reflect subclinical disease activity rather than only antecedent inflammatory risk. We also did not formally model competing risks, such as non‐cardiovascular death, which may be relevant in an older cohort. In addition, the methods used to pool *p* values for restricted cubic spline analyses and subgroup interaction terms were simplified and should be interpreted with this methodological consideration in mind.

### 4.5 Clinical and Public Health Implications

Our findings have several potential clinical and public health implications for the Chinese population, which faces a substantial and growing burden of cardiovascular disease [1, 2, 21]. The observed association between higher baseline hs‐CRP levels and MACE supports consideration of inflammatory status in cardiovascular risk assessment, particularly in the context of residual inflammatory risk [15]. At the same time, the findings from the log‐hsCRP two‐point change pattern analysis should be interpreted cautiously, because the overall association did not reach conventional statistical significance and the subgroup findings were exploratory. Accordingly, our results do not justify immediate changes in clinical practice, but they do support further evaluation of hs‐CRP as a complementary marker in cardiovascular risk stratification. In this context, evidence from trials such as JUPITER supports the relevance of inflammation as a therapeutic target in selected populations [22].

We did not observe a statistically significant independent association between baseline LDL‐C or LDL‐C two‐point change pattern groups and MACE in this comprehensively medication‐adjusted analysis. While LDL‐C remains a cornerstone of cardiovascular prevention and a primary target in guidelines, including the 2023 Chinese guideline for lipid management [4], our findings suggest that in populations with access to lipid‐lowering therapy, risk stratification based solely on LDL‐C may not fully capture cardiovascular risk. This underscores the need to interpret LDL‐C levels in the context of treatment status and other contributors to risk, including inflammation [13]. The finding that even one elevated biomarker, high LDL‐C or high hs‐CRP, was associated with increased MACE risk also supports the value of a broader multi‐marker perspective. Still, this should not be taken to diminish the importance of LDL‐C monitoring or lipid‐lowering therapy. The joint biomarker findings should also be interpreted cautiously, given the non‐significant estimate for the group with concurrent elevation of both biomarkers and the limited precision after subgrouping.

### 4.6 Future Research Directions

Future research should use longitudinal data with more frequent biomarker measurements, ideally three or more time points, to identify more refined and potentially non‐linear change patterns of LDL‐C and hs‐CRP. Detailed time‐varying information on medication use, including type, dosage, adherence, and duration, will also be important for separating biomarker‐related associations from treatment effects. It would also be valuable to investigate the genetic, lifestyle, and environmental determinants of different hs‐CRP change patterns, particularly factors associated with a favorable High baseline, decreasing pattern. In addition, future work could explore joint longitudinal models combining LDL‐C, hs‐CRP, and other relevant cardiovascular markers. Lipoprotein(a), for example, is an independent and genetically influenced risk factor for ASCVD and has been associated with MACE even among individuals with well‐controlled LDL‐C or across different hs‐CRP levels [23, 24, 25]. Assessing joint change patterns including Lp(a) may provide a more comprehensive picture of cardiovascular risk. Other lipid‐related markers such as small dense LDL‐C, the atherogenic index of plasma, or VLDL‐cholesterol, also warrant further study in longitudinal frameworks [26, 27]. Given potential ethnic differences in lipid metabolism and treatment response [14, 18, 28], external validation in other large and independent cohorts, particularly within diverse Asian populations, will be important.

## Author Contributions

Jiayin Guo, Bin Zhao, and Zongwei Liu contributed equally to this work as co‐first authors. Jiayin Guo, Bin Zhao, and Zongwei Liu conceived and designed the study, performed the data analysis, and drafted the main manuscript text. Zhian Liang analyzed the data and revised the manuscript. Yujun Shen and Xiangchen Dai co‐supervised the study, interpreted the results, and critically revised the manuscript for important intellectual content. Jiayin Guo and Bin Zhao prepared Figures 1, 2, 3, 4, 5, 6, 7 and Tables 1, 2, 3, 4, 5. All authors reviewed, edited, and approved the final manuscript for submission.

## Funding

The authors have nothing to report.

## Ethics Statement

The China Health and Retirement Longitudinal Study (CHARLS) was approved by the Institutional Review Board (IRB) of Peking University (Ethics number: IRB00001052–11015). The study was conducted in accordance with the principles of the Declaration of Helsinki. All participants provided written informed consent before an interview and a physical examination were conducted. For this study, we used publicly available, de‐identified data from the CHARLS, and therefore, no further ethical approval was required from our institution.

## Conflicts of Interest

The authors declare that they have no known competing financial interests or personal relationships that could have appeared to influence the work reported in this paper. All authors have reviewed the final manuscript and approved its submission.

## Transparency Statement

Xiangchen Dai affirms that this manuscript is an honest, accurate, and transparent account of the study being reported; that no important aspects of the study have been omitted; and that any discrepancies from the study as planned have been explained.

## Supporting information


**Table S1:** Proportion of Missing Data for Baseline Covariates Before Multiple Imputation (N = 9597).

## Data Availability

The data that support the findings of this study are available from the China Health and Retirement Longitudinal Study (CHARLS). These data are not directly available from the authors but can be obtained by eligible researchers through the official CHARLS platform, subject to the relevant data access procedures and terms of use.
